# Impact of Age on Biology, Presentation and Outcomes in Marginal Zone Lymphoma: Results From a Multicenter Cohort Study

**DOI:** 10.1002/hon.70087

**Published:** 2025-04-22

**Authors:** Pallawi Torka, Natalie S. Grover, Timothy J. Voorhees, Reem Karmali, Kaitlin Annunzio, Marcus P. Watkins, Andrea Anampa‐Guzmán, Heather Reves, Montreh Tavakkoli, Beth Christian, Colin Thomas, Stefan K. Barta, Praveen Ramakrishnan Geethakumari, Nancy L. Bartlett, Geoffrey Shouse, Adam J. Olszewski, Narendranath Epperla

**Affiliations:** ^1^ Department of Medicine Roswell Park Comprehensive Cancer Center Buffalo New York USA; ^2^ Department of Medicine Memorial Sloan Kettering Cancer Center New York New York USA; ^3^ Department of Medicine Lineberger Comprehensive Cancer Center University of North Carolina Chapel Hill North Carolina USA; ^4^ Division of Hematology Department of Medicine Ohio State University Comprehensive Cancer Center Columbus Ohio USA; ^5^ Department of Medicine Northwestern University Chicago Illinois USA; ^6^ Department of Medicine Siteman Cancer Center Washington University School of Medicine St. Louis Missouri USA; ^7^ Department of Medicine Harold C. Simmons Comprehensive Cancer Center UT Southwestern Medical Center Dallas Texas USA; ^8^ Department of Medicine University of Pennsylvania Philadelphia Pennsylvania USA; ^9^ Department of Medicine Thomas Jefferson University Philadelphia Pennsylvania USA; ^10^ Department of Medicine City of Hope National Medical Center Duarte California USA; ^11^ Department of Medicine Brown University Providence Providence Rhode Island USA; ^12^ Division of Hematology and Hematologic Malignancies Huntsman Cancer Institute University of Utah Salt Lake City Utah USA

**Keywords:** Elderly, marginal zone lymphoma, non‐Hodgkin lymphoma, overall survival, progression‐free survival

## Abstract

Given the paucity of age‐specific data about biology, presentation, and treatment outcomes in adults with MZL, we sought to evaluate differences between younger (≤ 70 years) and older (> 70 years) patients with MZL in a large retrospective cohort treated in the contemporary era (2010 onwards). The primary objective was progression‐free survival (PFS), while secondary objectives included the evaluation of overall survival (OS) and the cumulative incidence of transformation between the 2 groups. A total of 598 patients were included in the analysis and among these 32% were > 70 years of age. There were no age‐based differences in the prevalence of NMZL, SMZL, and EMZL. Older patients had a higher incidence of adverse prognostic features at diagnosis such as worse performance status, advanced stage disease, and bone marrow involvement, yet were more likely to be treated with single‐agent rituximab than chemoimmunotherapy. Age > 70 years was associated with inferior PFS and OS after controlling for clinically relevant risk factors and accounting for differences in first‐line treatment. Receipt of rituximab monotherapy was associated with significantly inferior PFS overall, however, the type of first‐line therapy did not impact OS in any group. Our data suggests that despite the development of new drugs for MZL, age remains an independent predictor of inferior outcomes. Investigation of targeted therapy combinations in the first‐line setting may yield the required balance of efficacy and toxicity in older adults with MZL.

## 1 Introduction

The incidence of all subtypes of marginal zone lymphoma (MZL) increases sharply with age [1] with the median age at diagnosis ranging between 65 and 70 years for splenic (SMZL), nodal (NMZL), and extranodal MZL (EMZL) of the mucosa‐associated lymphatic tissue (MALT) [2]. Pediatric nodal MZL is an extremely indolent disease classified as a separate entity in the fifth edition of The World Health Organization (WHO) Classification of Haematolymphoid Tumours [3]; however, age‐specific data on the biology of MZL, clinical presentation, and treatment outcomes in adults are otherwise lacking.

Advancing age was associated with a shorter lymphoma specific survival and overall survival (OS) in all MZL subtypes in a SEER database study [4], however differences in therapy were not accounted for in the analysis. There are many treatment options in MZL with no known differences in survival, and choice of therapy is commonly based on data from phase 2 trials which are subject to enrollment bias. These results may be less relevant to patients in the ‘real‐world’ who are older and less fit in general [5]. Herein, we evaluated the impact of age on biology, clinical presentation, and outcomes in patients with MZL utilizing a large, multicenter, retrospective cohort.

## 2 Patients and Methods

### 2.1 Study Design

This retrospective, multicenter study included adults with MZL treated with first‐line systemic therapy at 10 US medical centers on or after Jan 2010. The study was approved by the institutional review boards of all participating sites and was conducted in compliance with the Declaration of Helsinki.

We collected variables known to be significantly associated with survival outcomes in all subtypes of MZL. Values of laboratory tests (albumin, hemoglobin [hb], serum lactate dehydrogenase (LDH), and β‐2‐microglobulin) were harmonized based on the upper or lower limit of normal at each institution. All staging procedures (e.g., bone marrow evaluations) and treatment evaluations were conducted per local practice.

### 2.2 Ethics Approval and Consent to Participate

The study was approved by the institutional review board at Ohio State University and at all participating sites and was conducted in compliance with the Declaration of Helsinki. As this was a retrospective study, informed consent was waived.

### 2.3 Study Objectives and Definitions

Patients were divided into 2 groups based on age at the start of first‐line systemic therapy ≤ 70 years and > 70 years. The age cutoff of 70 years was based on the prior literature (MALT‐IPI [6]), which was also validated as an appropriate cut‐off for analysis in our dataset (Figure S1). The relationship between survival outcomes was non‐linear, with a sharp increase in risk after age 70. We further observed that the choice of treatment (rituximab monotherapy rather than a combination with chemotherapy) depended on age, with a strong preference for chemotherapy‐free approach in patients older than 70 (Figure S2).

The primary endpoint was progression‐free survival (PFS) in these 2 groups. PFS was defined as the time from the start of first‐line therapy to lymphoma relapse, progression, death from any cause, or censoring at the last clinical assessment. The secondary objectives included the evaluation of OS and the cumulative incidence of transformation between the 2 groups.

### 2.4 Statistical Analysis

Demographic and disease characteristics were summarized using medians and ranges for continuous variables and frequencies and percentages for categorical variables. They were compared among study groups using the rank sum test for continuous variables and Fisher exact test for categorical variables. PFS was estimated using the Kaplan‐Meier method and compared between groups using the log‐rank test. Cox proportional hazard regression models were used to estimate the hazard ratios (HRs) for risk of progression or death. The multivariable Cox model was built adjusting for a priori selected confounders of known clinical relevance: sex, performance status, MZL histologic subtype, stage, presence of B‐symptoms, presence of high (> 20%, a cutoff established in our prior study [7]) Ki‐67 staining on the biopsy, low serum albumin, and high serum LDH, with additional stratification based on first‐line therapy regimen. The proportional hazard assumption was verified using Schoenfeld residuals after fitting Cox models. All multivariable models used multiple imputation by chained equations [8], to mitigate potential bias related to data missing at random on performance status (missing in 13%), B symptoms (5%), albumin (11%), and LDH (13%). Model coefficients from 50 imputed datasets were averaged using Rubin's rules. The analysis of cause‐specific deaths and of the histologic transformation was conducted using the competing‐risk framework, plotting cumulative incidence function, and comparing groups using Gray's test and competing risk models. OS was calculated from the start of first‐line treatment and compared using the log‐rank test. Analyses were performed using Stata version 17 (StataCorp, College Station, TX), and all statistical tests were 2‐sided, with a type‐1 error < 0.05 indicating statistical significance. All estimates were reported with 95% confidence intervals (95% CIs).

## 3 Results

### 3.1 Baseline Characteristics

This study included 598 MZL patients of which 405 (68%) were ≤ 70 years and 193 (32%) were > 70 years old (including 10% who were > 80 years old) at the start of first line systemic therapy. The median age was 59 years in ≤ 70 years group, while it was 77 years in > 70 years cohort. About half of the patients had EMZL (49%), and a quarter each had NMZL (27%) and SMZL (25%). Ninety‐two percent had an ECOG performance status (PS) of 0–1, 81% had advanced stage disease, and 54% had bone marrow involvement. Table 1 shows the baseline characteristics categorized by age (≤ 70 years and > 70 years). Patients aged > 70 years presented more often with ECOG PS ≥ 2 (13% vs. 6%), stage 3–4 disease (88% vs. 78%), bone marrow involvement (64% vs. 50%), and M protein at diagnosis (51% vs. 38%) than those ≤ 70 years. Median follow‐up in the entire cohort was 5.2 years (95% CI = 5.0–5.5 years), and it was 5.5 years for patients aged ≤ 70 and 4.8 years for those aged > 70 years (log‐rank *p* = 0.13).

**TABLE 1:** Baseline patient and disease characteristics.

	All patients *N* = 598 (%)	≤ 70 years *n* = 405 (%)^a^	> 70 years *n* = 193 (%)^a^
Gender			
Male	293 (49)	194 (48)	99 (51)
Female	305 (51)	211 (52)	94 (49)
MZL subtype			
NMZL	159 (27)	100 (25)	59 (31)
SMZL	149 (25)	97 (24)	52 (27)
EMZL	290 (48)	208 (51)	82 (43)
ECOG PS			
0–1	478 (92)	331 (94)	147 (87)
≥ 2	42 (8)	20 (6)	22 (13)
Stage			
1–2	114 (19)	91 (23)	23 (12)
3–4	484 (81)	314 (78)	170 (88)
BM involvement			
No	212 (46)	164 (50)	48 (36)
Yes	251 (54)	164 (50)	87 (64)
Not done	135	77	58
B symptoms			
No	461 (81)	313 (81)	148 (80)
Yes	109 (19)	73 (19)	36 (20)
LDH > ULN^b^			
No	378 (73)	259 (73)	119 (72)
Yes	140 (27)	94 (27)	46 (28)
WBC (K/uL), median (range)	6.20 (0.7–151)	6.11 (0.7–115.3)	6.3 (1.2–151)
Hb (g/dL), median (range)	12.4 (3.7–18.9)	12.5 (4.7–18.9)	12.3 (3.7–16.2)
Serum albumin			
Normal	463 (87)	315 (88)	148 (85)
Low^b^	69 (13)	44 (12)	25 (15)
M‐protein	127 of 302 (42)	75 of 200 (38)	52 of 102 (51)
TP53 alteration	16 of 256 (6)	8 of 171 (2)	8 of 85 (4)
Complex cytogenetics	23 of 302 (8)	16 of 208 (8)	7 of 94 (7)
CD5 expression			
No	463 (88)	307 (86)	156 (92)
Yes	63 (12)	50 (14)	13 (8)
Ki67%			
≤ 20%	223 (75)	149 (75)	74 (75)
> 20%	75 (25)	51 (25)	24 (25)
Unrecorded	300	205	95
First‐line therapy			
Rituximab	353 (59)	222 (55)	131 (68)
BR	186 (31)	141 (35)	45 (23)
RCHOP	39 (7)	30 (7)	9 (5)
RCVP	20 (3)	12 (3)	8 (4)
Median DTI in days (range)	55 (1–4071)	54 (1–2729)	57 (1–4071)

Abbreviations: BM, bone marrow; BR, rituximab; bendamustine; DTI, Diagnosis to treatment interval; ECOG PS, Eastern Cooperative Oncology Group Performance Status; EMZL, Extranodal marginal zone lymphoma; LDH, Lactate Dehydrogenase; NMZL, Nodal marginal zone lymphoma; R‐CHOP, rituximab, cyclophosphamide, doxorubicin, vincristine, prednisone; RCVP, rituximab, cyclophosphamide, vincristine, prednisone; SMZL, Splenic marginal zone lymphoma; ULN, Upper limit of normal; WBC, White blood cell.

^a^ Percentages based on total number of patients with available data.

^b^ Based on institutional standard.

The most common first‐line treatment was rituximab monotherapy (*n* = 353, 59%), followed by rituximab and bendamustine (BR) in 31% (*n* = 186) of patients and rituximab, cyclophosphamide, vincristine, prednisone +/− doxorubicin (R‐CHOP/R‐CVP) in a minority of patients (*n* = 59, 10%). Patients > 70 years were more likely to be treated with rituximab monotherapy compared to those ≤ 70 years (68% vs. 55%; Figure S2) and less likely to receive BR (23% vs. 35%). The median time from diagnosis to treatment initiation was 55 days in the entire cohort and was comparable between the 2 groups (57 vs. 54 days).

### 3.2 Progression‐Free Survival

**FIGURE 1:**
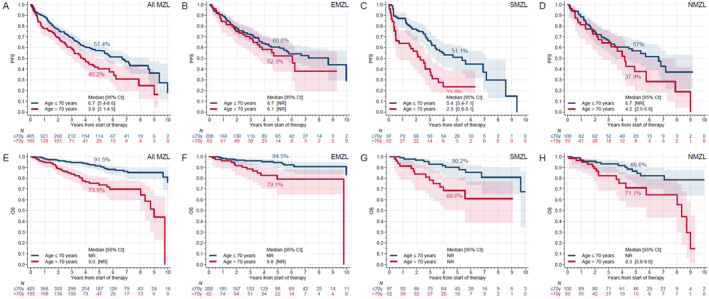
Impact of age on progression free survival (panels A–D) and overall survival (panels E–H) in all patients and subtypes of marginal zone lymphoma.

The median PFS (from the start of first‐line systemic therapy) for patients aged > 70 years was significantly inferior compared to those aged ≤ 70 years (3.8 vs. 6.7 years, *p* = 0.001, Figure 1A). When evaluated by MZL subgroup among patients aged > 70 years versus ≤ 70 years, median PFS was significantly inferior in SMZL (2.3 vs. 5.4 years, < 0.001, Figure 1C) but not in the EMZL (6.1 vs. 8.7 years, *p* = 0.35, Figure 1B) or NMZL (4.2 vs. 6.7 years, *p* = 0.15, Figure 1D). After adjusting for gender, MZL subtype, ECOG PS, stage, B symptoms, Hb, Ki‐67%, albumin, LDH and type of first‐line treatment in the multivariable analysis, age > 70 years remained associated with a shorter PFS (aHR = 1.38, 95% CI = 1.04–1.82, *p* = 0.026) (Table S1).

**FIGURE 2:**
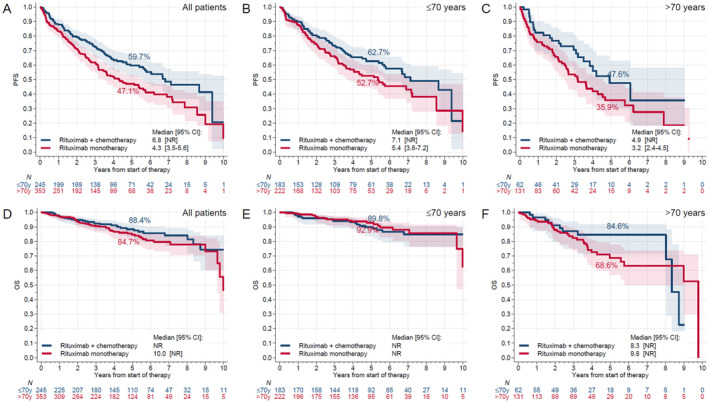
Impact of first line therapy on progression free survival (panels A–C) and overall survival (panels D–F) in patients with marginal zone lymphoma.

We then evaluated the impact of first‐line therapy on PFS. Use of rituximab monotherapy was associated with a shorter median PFS compared to chemoimmunotherapy (BR/RCVP/RCHOP) in the entire cohort (4.3 vs. 6.8 years, *p* = 0.002, Figure 2A). When evaluated by age, patients aged ≤ 70 years had a shorter median PFS among those who received rituximab monotherapy compared to chemoimmunotherapy (5.4 vs. 7.1 years, *p* = 0.04, Figure 2B), but the difference did not reach statistical significance in patients aged > 70 years (3.2 vs. 4.9 years, *p* = 0.05, Figure 2C). In multivariable models, PFS remained significantly shorter for all patients treated with rituximab monotherapy (aHR = 1.59, 95% CI = 1.20–2.10, *p* = 0.001). A similar finding was noted for those aged ≤ 70 years treated with rituximab monotherapy (aHR = 1.68, 95% CI = 1.19–2.36, *p* = 0.003) in multivariable analysis, however, the difference was not statistically significant for those aged > 70 years (aHR = 1.23, 95% CI = 0.73–2.08, *p* = 0.43; Table S2).

### 3.3 Overall Survival

The median OS (from the start of first‐line systemic therapy) for patients aged > 70 years was significantly inferior compared to those aged ≤ 70 years (9 years vs. not reached [NR], *p* < 0.001, Figure 1E). Median PFS was significantly inferior in patients aged > 70 years compared to those aged ≤ 70 years across all MZL subgroups (Figure 1F–H). After adjusting for gender, MZL subtype, ECOG PS, stage, B symptoms, Hb, Ki67%, albumin, LDH and type of first‐line treatment in the multivariate analysis, age > 70 years remained associated with a significantly shorter OS in the entire cohort (aHR = 3.06, 95% CI = 1.88–4.97, *p* < 0.001) (Table S3). There were no differences in OS based on the type of first line therapy in the entire cohort or when stratified by age (Figure 2D–F, Table S4).

### 3.4 Cumulative Incidence of Histologic Transformation (HT)

**FIGURE 3:**
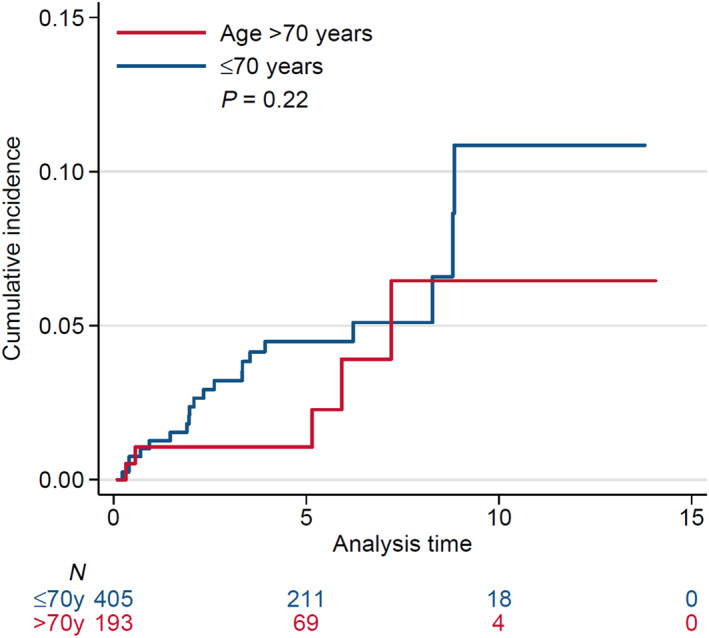
Cumulative incidence of transformation in patients with marginal zone lymphoma stratified by age group.

Twenty‐five HT events occurred in the study population (*n* = 598), five in patients aged > 70 years and 20 in those aged ≤ 70 years. The cumulative incidence of HT was 3.4% at 5 years (95% CI = 2.1–5.3) and 9.2% at 10 years (95% CI = 5–14.8). The difference in the cumulative incidence of HT (from the MZL diagnosis) between the > 70 years and ≤ 70 years groups was not statistically significant, with 5‐ and 10‐year rates of transformation of 1.1% versus 4.5% and 6.5% versus 10.9%, respectively (Gray's test, *p* = 0.22, Figure 3).

### 3.5 Cause of Death

**FIGURE 4:**
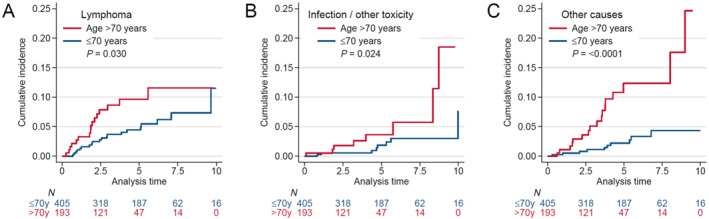
Cumulative incidence of death in patients with marginal zone lymphoma stratified by age group.

The most common cause of death in the entire group was lymphoma progression (*n* = 36) with 14 deaths related to infection, 2 secondary to other treatment toxicity, and 28 due to other causes deemed unrelated to the lymphoma or its treatment. The breakdown of cause of death by age (≤ 70 years and > 70 years) is shown in Table S5. The cumulative incidence of lymphoma specific mortality at 5 years was 4.5% (95% CI = 2.6–7.1) in patients aged ≤ 70 years compared to 9.7% (95% CI = 5.6–15) in those > 70 years (Gray's test, *p* = 0.03, Figure 4A), while the cumulative incidence of death due to toxicity or infection at 5 years was 1.9% (95% CI = 0.7–4.2) versus 3.6% (95% CI = 1.3–7.9), respectively (Gray's test, *p* = 0.024, Figure 4B). The cumulative incidence of death from other causes at 5 years was 2.2% (95% CI = 1.0–4.3) in patients aged ≤ 70 years compared to 12.4% (95%CI = 7.0–19.4) in those > 70 years (Gray's test, *p* < 0.001, Figure 4C).

## 4 Discussion

In this large retrospective cohort of newly diagnosed MZL patients, patients > 70 years of age presented more often with adverse prognostic features [6, 9, 10] such as worse performance status, advanced stage disease and bone marrow involvement and were more likely to be treated with rituximab monotherapy rather than chemoimmunotherapy. There were no age‐based differences in prevalence of EMZL, SMZL and NMZL. Age > 70 years was associated with significantly inferior PFS and OS after controlling for clinically relevant factors and accounting for differences in first‐line treatment with the impact of age being most pronounced in SMZL. Receipt of rituximab monotherapy was associated with significantly inferior PFS overall, however, the type of first‐line therapy did not impact OS in any group.

Advancing age was previously shown to be associated with inferior lymphoma‐specific survival in patients with MZL (diagnosed between 1995 and 2009) in a population based study using a Surveillance, Epidemiology, and End Results (SEER) data base [4]. The MALT‐IPI was developed based on data from patients with MALT lymphoma treated in the international randomized International Extranodal Lymphoma Study Group 19 (IELSG‐19) trial with rituximab, chlorambucil or rituximab plus chlorambucil, and validated in 3 independent cohorts who were treated before the use of BR became commonplace [6]. The MALT‐IPI includes age ≥ 70 years, Ann Arbor stage III or IV, and an elevated LDH to identify 3 groups, low, intermediate, and high risk (corresponding to the presence of 0, 1, or ≥ 2 of these factors, respectively) that have a 5‐year event free survival (EFS) rates of 70%, 56%, and 29%, respectively. Subsequently, a SEER data base analysis of patients with localized EMZL demonstrated age ≥ 60 years as an independent factor associated with shorter lymphoma‐specific survival [11], which was corroborated on revised MALT‐IPI [9]. Our study confirms that age remains an important prognostic variable in the era of contemporary regimens such as BR. While age> 60 years was originally used in many prognostic indices, with the shifting demographics, higher age cut offs are increasing being used to define ‘older adults (OA)’ in recent studies in lymphoma [12, 13, 14]. We examined outcomes of patients according to age ≤ 70 and > 70 years, choosing this cutoff based on prior knowledge (as established by MALT‐IPI) and on the empiric basis, using observed outcomes and the general clinical considerations related to known geriatric comorbidities and other factors that determine treatment choices for MZL. Of note, this approach is different than in prognostic models which strive for individual accuracy of prediction, where modeling age as a continuous (or at least more granular) variable is important to capture potential non‐linear associations between outcomes and age [15, 16]. We note that age > 70 (although statistically significant with a PFS HR of 1.71) was not selected as an independent variable in the recently developed MZL‐IPI which is applicable to all subtypes of MZL and relies primarily on laboratory parameters (serum LDH, hemoglobin, absolute lymphocyte count and platelet count) and nodal or disseminated MZL presentation [17]. We found that age > 70 strongly correlated with hemoglobin and stage 4 disease, but not with LDH level, however our dataset lacked the information on lymphocyte or platelet counts, so we could not validate the MZL‐IPI. Because age > 70 years was an important predictor of treatment with rituximab monotherapy as well as cause‐specific mortality, particularly from causes unrelated to lymphoma or treatment toxicity, it is important to consider it as a factor in clinical decision‐making, as the specific risks and preferences of OA may differ from younger individuals.

Since most studies reporting impact of age and other baseline characteristics are conducted retrospectively using patient level data or large databases, the impact of geriatric syndromes and baseline frailty has not been studied in MZL [2]. In our cohort, age > 70 years was associated with inferior PFS and OS even after controlling for baseline disease characteristics and type of therapy. We have previously also reported that age > 70 years is associated with POD24 [18], an additional marker of poor MZL disease biology which may not be captured with other baseline variables [19]. Inferior disease specific responses could result from more aggressive disease biology in OAs, however that is unlikely to be the case since markers of aggressive biology such as Ki‐67^7^, *TP53* mutations and complex cytogenetics were similar between the younger and older patients, with the caveat these data were missing for a large proportion of patients. Another possibility is that increased adverse effects of therapy in a subset of OAs led to premature treatment discontinuation and shorter PFS which makes it even more imperative to assess impact of comorbidity, polypharmacy and geriatric syndromes on treatment outcomes. These variables can be combined with disease biology to better inform therapeutic decisions in OA with MZL to allow for a truly personalized approach. While advancing age can certainly contribute to an inferior OS due to competing non‐lymphoma mortality, other contributing factors such as ineligibility or intolerance to subsequent lines of therapy are likely to be at play.

We also noted that patients > 70 years presented more often with adverse prognostic markers such as worse performance status, advanced stage disease and bone marrow involvement. Presentation at a more advanced stage could be due to a more aggressive disease biology or lack of access to care leading to delays in diagnosis and treatment. Due to missing data on Ki67, *TP53* mutations, complex cytogenetics and M protein at diagnosis (and potential bias in testing by clinicians), we could not conclusively evaluate the age‐based differences between disease biology; however, with the exception of presence of M protein at diagnosis, these were similar between the younger and OAs in the subset of patients with available data. This is not surprising since monoclonal gammopathy of undetermined significance (MGUS) increases in incidence with age [20].

OAs were more likely to be treated with single agent rituximab rather than chemoimmunotherapy. Receipt of single agent rituximab was associated with inferior PFS in the entire cohort and more specifically in patients ≤ 70 years of age, with minor differences noted in older adults. This suggests that all older adults may not benefit from more aggressive therapy. However, fit OAs should be considered for chemoimmunotherapy in appropriate scenarios such as symptomatic bulky disease due to its ability to achieve deeper responses. Geriatric assessment (GA) is a systematic and validated approach for identifying strengths and treating vulnerabilities of OAs with cancer. GA results can inform treatment decision making and guide interventions to mitigate vulnerabilities. Previous studies have shown that a formal GA outperforms physician's clinical judgement and ECOG performance status in identifying frailty [21, 22] and is recommended by the American Society of Clinical Oncology in OA undergoing systemic cancer therapy [23]. Data regarding the utility of GA in patients with lymphoma, especially MZL, is limited, and prospective studies are needed to understand its impact on therapeutic decision making and outcomes. Type of first line therapy did not impact overall survival which is consistent with previous studies [24, 25] since many patients who have suboptimal response to first line rituximab subsequently receive chemoimmunotherapy or, increasingly, other salvage therapies such as BTK inhibitors with good benefit.

Limitations of our study include its retrospective nature and selection bias that is inherently present in studies using data from selected centers. We could not ascertain age‐based differences in disease biology due to paucity of molecular and cytogenetic data obtained in routine clinical practice. Geriatric assessments are still rare in clinical practice, so decisions about therapy in our cohort were likely made based on subjective estimates of patient frailty and ability to tolerate chemotherapy.

In summary, patients with MZL > 70 years of age presented with more advanced stage disease and had inferior PFS and OS compared to their younger counterparts even after adjusting for other prognostic variables and type of frontline therapy. Assessment of baseline fitness and geriatric vulnerabilities is required to evaluate how aggressive disease biology and lower functional reserve contribute to the inferior prognosis.

## Author Contributions

Conception and design: N.E. and P.T. Financial support: None. Collection and assembly of data: All authors. Data analysis: A.J.O. and N.E. Interpretation: All authors. Manuscript writing: First draft prepared by P.T. All authors provided critical and insightful comments. Final approval of manuscript: All authors.

## Ethics Statement

The study was approved by the Institutional Review Board (IRB) of Ohio State University (OSU). The study conforms to the Declaration of Helsinki.

## Consent

A waiver of consent was obtained from IRB at OSU. The waiver was obtained because of the retrospective nature of the study with minimal risk to the subjects and that many of the patients either passed away or had lost to follow‐up since their treatment.

## Conflicts of Interest

P.T.: Honoraria/consulting/ad boards for Seagen, TG Therapeutics, ADC Therapeutics, Genentech, GenMab and Lilly USA. N.S.G.: Research funding: Tessa Therapeutics, Honoraria/consulting/ad boards for ADC Therapeutics, Genentech, Kite, Novartis, Ono Pharma, BMS, Regeneron, Janssen, Caribou, and Seagen. R.K.: Advisory Board: BMS, Miltenyi, Abbvie, Genmab, Lilly USA, Genentech/Roche; Grants/Research Support: BMS. Speakers Bureau: AstraZeneca, BeiGene, Morphosys/Incyte. B.C.: Research funding: Genentech, Acerta, Millenium, Bristol‐Myers Squibb, F Hoffman‐La Roche; Advisory Board: Incyte, AstraZeneca. S.K.B.: Honoraria: Acrotech, Affimed, Daiichi Sankyo, Kyowa Kirin, Janssen, Seagen. P.R.G.: Consultancy services: Kite/Gilead Pharma, Bristol Myers Squibb (BMS). Advisory board: Pharmacyclics LLC, ADC Therapeutics, Cellectar Biosciences, Ono Pharma, CRISPR therapeutics, IPSEN Biopharma, and Regeneron Pharmaceuticals. N.L.B.: Research funding: ADC Therapeutics, Autolus, BMS, Celgene, Forty Seven, Genentech, Immune Design, Janssen, Merck, Millennium, Pharmacyclics, Affirmed Therapeutics, Dynavax, Gilead, MedImmune, Novartis; Consulting/Ad board: Kite Pharma, Pfizer, ADC Therapeutics, Roche/Genentech, Seattle Genetics, BTG, Acerta. A.J.O.: Genmab; Precision Bio; Adaptive Biotechnologies; Celldex; Acrotech Biopharma; Schrodinger; TG Therapeutics; Genentech. N.E.: Research funding: Beigene, Lilly, and Incyte; Speakers Bureau for Beigene and Genetech, Ad boards for ADC Therapeutics; Honararium from Novartis. T.J.V.: Research Funding: Morphosys, Incyte, Genmab, AbbVie, Viracta, Kite Pharma, Recordati. Consultancy: Novartis, Recordati, AbbVie. Ad Boards for AbbVie.

### Peer Review

The peer review history for this article is available at https://www.webofscience.com/api/gateway/wos/peer-review/10.1002/hon.70087.

## Supporting information

Supporting Information S1

## Data Availability

De‐identified data will be made available on request to the corresponding author should the request meet the regulatory requirement per institutional IRB.
