# Supplementary material for: Impact of Age on Biology, Presentation and Outcomes in Marginal Zone Lymphoma: Results From a Multicenter Cohort Study
Source: Hematol Oncol. 2025 Apr 22;43(3):e70087. doi: 10.1002/hon.70087 (PMC12013245; doi:10.1002/hon.70087)
Supplement: Supplementary file 1 — Supporting Information S1 [file HON-43-e70087-s001.docx]

**SUPPLEMENTAL APPENDIX**

**Table of Contents** 1

**Impact of age on PFS:** Table S1 2

**Impact of first-line therapy on PFS:** Table S2 3

**Impact of age on OS:** Table S3 4

**Impact of first-line therapy on OS:** Table S4 5

**Causes of Death:** Table S5 6

**Relation between survival outcomes and age:** Figure S1 7

**Relation between treatment preference and age:** Figure S2 8

**Table S1: Association between age** **(≤70 yrs vs >70 yrs) and progression free survival**

| **MZL groups** | **Univariable** | | **Multivariable*** | |
| --- | --- | --- | --- | --- |
|  | **HR (95% CI)** | **p-value** | **HR (95% CI)** | **p-value** |
| All patients | 1.54 (1.18-2.00) | 0.001 | 1.38 (1.04-1.82) | 0.026 |
| SMZL | 2.26 (1.41-3.61) | <0.001 | 2.24 (1.29-3.88) | 0.004 |
| EMZL | 1.22 (0.80-1.87) | 0.35 | 0.98 (0.62-1.56) | 0.94 |
| NMZL | 1.43 (0.87-2.35) | 0.15 | 1.09 (0.60-2.01) | 0.77 |

Abbreviations: MZL- Marginal zone lymphoma, SMZL- Splenic marginal zone lymphoma, EMZL- Extranodal marginal zone lymphoma, NMZL- Nodal marginal zone lymphoma

*Multivariable model included age, gender, MZL subtype, ECOG PS, stage, B symptoms, Hb, Ki67%, albumin, LDH

**Table S2: Association between first line therapy (rituximab monotherapy vs** **chemoimmunotherapy) and progression free survival**

| **Age groups** | **Univariable** | | **Multivariable*** | |
| --- | --- | --- | --- | --- |
|  | **HR (95% CI)** | **p-value** | **HR (95% CI)** | **p-value** |
| All patients | 1.51 (1.17-1.95) | 0.002 | 1.59 (1.20-2.10) | 0.001 |
| ≤70 yrs | 1.39 (1.01-1.90) | 0.041 | 1.68 (1.19-2.36) | 0.003 |
| >70 yrs | 1.60 (1.00-2.56) | 0.05 | 1.23 (0.73-2.08) | 0.43 |

*Multivariable model included age, gender, MZL subtype, ECOG PS, stage, B symptoms, Ki67%, albumin, LDH

**Table S3. Association between age (≤70 yrs vs >70 yrs) and overall survival**

| **MZL groups** | **Univariable** | | **Multivariable*** | |
| --- | --- | --- | --- | --- |
|  | **HR (95% CI)** | **p-value** | **HR (95% CI)** | **p-value** |
| All patients | 3.38 (2.17-5.29) | <0.001 | 3.06 (1.88-4.97) | <0.001 |
| SMZL | 3.34 (1.49-7.51) | 0.004 | 5.02 (1.66-15.13) | 0.004 |
| EMZL | 3.50 (1.58-7.76) | 0.002 | 2.92 (1.23-6.91) | 0.015 |
| NMZL | 2.72 (1.31-5.65) | 0.007 | 1.97 (0.79-4.94) | 0.15 |

Abbreviations: MZL- Marginal zone lymphoma, SMZL- Splenic marginal zone lymphoma, EMZL- Extranodal marginal zone lymphoma, NMZL- Nodal marginal zone lymphoma

*Multivariable model included age, gender, MZL subtype, ECOG PS, stage, B symptoms, Hb, Ki67%, albumin, LDH

**Table S4. Association between first line therapy (rituximab monotherapy vs chemoimmunotherapy) and overall survival**

| **Age groups** | **Univariable** | | **Multivariable*** | |
| --- | --- | --- | --- | --- |
|  | **HR (95% CI)** | **p-value** | **HR (95% CI)** | **p-value** |
| All patients | 1.41 (0.89-2.21) | 0.14 | 1.35 (0.82-2.23) | 0.24 |
| ≤70 yrs | 0.96 (0.51-1.81) | 0.89 | 1.21 (0.60-2.42) | 0.59 |
| >70 yrs | 1.41 (0.71-2.81) | 0.33 | 1.19 (0.53-2.68) | 0.67 |

*Multivariable model included age, gender, MZL subtype, ECOG PS, stage, B symptoms, Ki67%, albumin, LDH

**Table S5. Causes of Death**

| **Cause of death** | **All**  **N=80 (%)** | **≤70 years**  **n=38 (%)** | **>70 years**  **N=42 (%)** |
| --- | --- | --- | --- |
| Lymphoma progression | 36 (45) | 20 (53) | 16 (38) |
| Infection | 14 (18) | 7 (18) | 7 (17) |
| Toxicity | 2 (2) | 1 (3) | 1 (2) |
| Other causes* | 28 (35) | 10 (26) | 18 (43) |

*This category includes causes of death that does not fit into one of the other listed categories.

**Figure S1. Non-linear relationship between survival outcomes and age**.

Panels show the relationship between exponentiated linear predictor for PFS **(A)** or OS **(B)** in survival models using age as a continuous variable specified using a restricted cubic spline. For both survival estimates, a sharp increase in the risk is observed beyond age 70.

Panels **(C) and (D)** show hazard ratios (HR) and Harrell’s C statistics from Cox models for PFS (C) or OS (D) using age dichotomized at different cutoffis. In this analysis in panel (C), the lowess smoother of HR plot shows an elbow around age 70, whereas the C-statistic increases linearly without a clear preferred cutoff. In the analysis in panel (D), the HR increases steadily from age 60 until 80, but C-statistic shows no further increase after the age of about 70.

**Figure S2. Non-linear relationship between treatment selection and age**.

Odds ratio (OR) for the use of single-agent rituximab versus combination with chemotherapy, adjusted for sex, MZL subtype, and stage of the lymphoma, have been calculated for each age cutoff, and plotted together with the corresponding Akaike information criterion (AIC) for the resulting model. The OR smoothed curve shows an inflection point around the age of 70.
